# Two Cases of Sarcoma Arising in Giant Cell Tumor of Bone Treated with Denosumab

**DOI:** 10.1155/2015/767198

**Published:** 2015-12-22

**Authors:** Cory Julian Broehm, Erika L. Garbrecht, Jeff Wood, Therese Bocklage

**Affiliations:** ^1^Department of Pathology, University of New Mexico School of Medicine, MSC08 4640, 1 University of New Mexico, Albuquerque, NM 87131, USA; ^2^Department of Orthopaedics, University of New Mexico School of Medicine, MSC10 5600, Albuquerque, NM 87131, USA; ^3^Department of Radiology, University of New Mexico School of Medicine, MSC10 5530, 1 University of New Mexico, Albuquerque, NM 87131, USA

## Abstract

Giant cell tumor (GCT) of bone is a generally benign, but often locally aggressive, neoplasm of bone, with a propensity for recurrence. Sarcomatous transformation is rare and typically occurs with a history of recurrences and radiation treatment. Denosumab, an inhibitor of the RANK ligand involved in bone resorption in GCT, is increasingly used in treatment of recurrent or unresectable giant cell tumor of bone. We report two cases of sarcomatous transformation of GCT to osteosarcoma in patients receiving denosumab. One was a 59-year-old male with a 12-year history of GCT and multiple recurrences taking denosumab for 2.5 years. The second case was in a 56-year-old male with a seven-year history of GCT taking denosumab for six months. Review of the literature shows one case report of malignant transformation of GCT in a patient being treated with denosumab. As the use of denosumab for treatment of GCT will likely increase, larger, controlled studies are needed to ascertain whether denosumab may play a role in malignant transformation of giant cell tumor of bone.

## 1. Introduction

Giant cell tumor (GCT) of bone is a generally benign tumor that is often locally aggressive, causing significant destruction of bone [1]. Recurrence may be seen in 15–50% of cases after treatment, usually within 2 years, depending on the location of the tumor and treatment modality [2–5]. Pulmonary metastasis may occur in less than 5% of cases [2, 4, 6]. Malignant transformation of GCT is rare, occurring in less than one percent of cases [1]. Secondary transformation, which follows radiation therapy or less commonly surgical intervention, accounts for approximately 70% of malignant GCT [7, 8]. Primary malignant GCT, which arise de novo alongside typical GCT, make up the remainder of malignant cases [7–9].

Treatment often involves curettage, with or without bone filler or adjuvants such as polymethylmethacrylate (PMMA) or phenol. Less invasive procedures, such as radiotherapy, radiofrequency thermal ablation, or chemoembolization, may be used in cases where surgery is not possible. Wide resection may be reserved for cases in which surgery results in relatively minor functional impairment or for tumors with extensive local destruction [2, 3, 10]. However, considerable morbidity may be involved in resection. Giant cell tumor is composed of neoplastic mononuclear stromal cells and reactive nonneoplastic multinucleated giant cells that are responsible for bone resorption, which is mediated by interaction between receptor activator of nuclear factor-kB (RANK) expressed by giant cells and RANK ligand (RANKL) on stromal cells [11]. Denosumab, a monoclonal antibody inhibitor of RANKL, has proven effective in limited clinical trials in halting tumor progression in patients with recurrent or unresectable giant cell tumors [12, 13].

A recent report described a case of high grade sarcoma arising in a giant cell tumor of bone treated with denosumab [14]. We report an additional two cases of high grade sarcoma arising in giant cell tumor of bone in patients receiving denosumab.

## 2. Case Reports

### 2.1. Case  1

**Figure 1:**
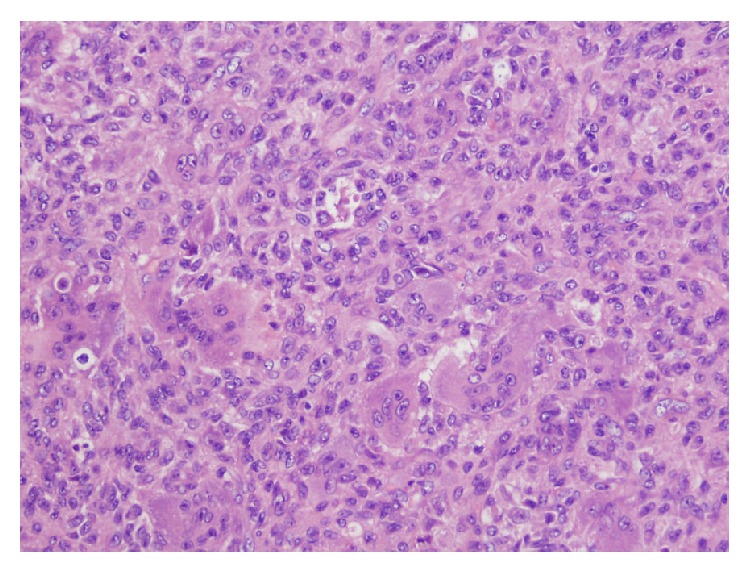
Histology of hemipelvectomy specimen showed mononuclear cells with interspersed multinucleated cells (hematoxylin and eosin stain, 20x).

A 46-year-old male presented in 2002 to an outside institution with five to six years of right hip pain radiating down the lateral thigh. Initial imaging is unavailable but by report radiographs showed a large mass involving the ischial tuberosity and portions of the adjacent inferior and superior rami of the right pelvis. He subsequently transferred care to our institution. Biopsies performed at the outside institution were reviewed and confirmed giant cell tumor of bone. He subsequently underwent partial internal hemipelvectomy in November 2002. Histopathologic analysis showed GCT (Figure 1).

Postoperatively, the patient was started on alendronate (dose unknown). Follow-up MRI in February 2003 showed an enhancing, high T2 signal, 2 cm soft tissue mass within the right adductor musculature, adjacent to the former location of the ischial tuberosity, suspicious of recurrence. CT imaging in August 2003 showed a mass within the surgical bed, within the obturator externus and pectineus muscles, and abutting the root of the penis. CT guided biopsy confirmed recurrent benign GCT. Reexcision of the mass was performed in September 2003. The pathology specimen showed a 4 cm mass embedded within excised soft tissue. Giant cell tumor was present at multiple resection margins.

**Figure 2:**
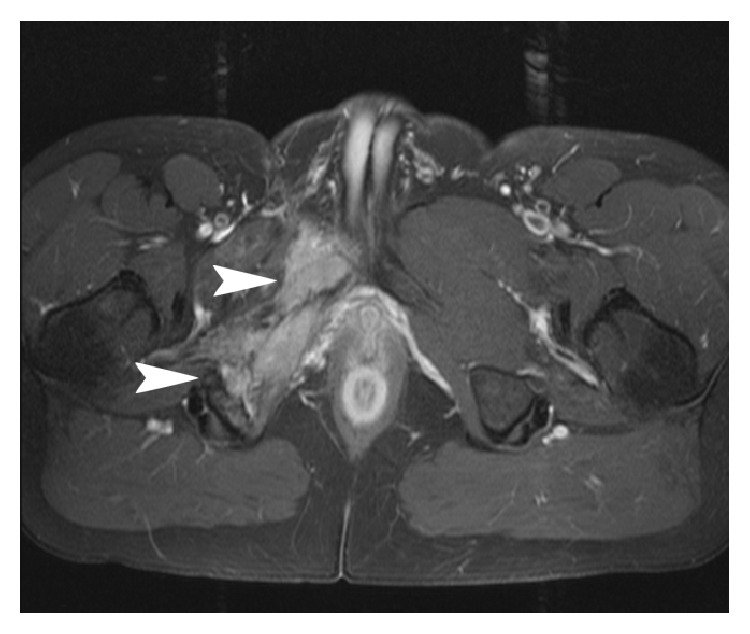
Gadolinium enhanced axial T1 CT (December 2005) of the pelvis with fat saturation demonstrates postsurgical change and irregularity of the right inferior pubic ramus, with an adjacent associated enhancing soft tissue mass (arrows).

Postoperatively, the patient did well, with intermittent complaints of pain. Follow-up MRI eight months after surgery showed an enhancing multilobulated mass at the margins of the original hemipelvectomy surgical bed, the largest mass measuring up to 2.7 cm. Edema and abnormal enhancement were noted within the obturator externus, obturator internus, and quadratus femoris muscles. Another 1.5 cm nodule was noted in the proximal adductor brevis muscle. He was subsequently followed up with approximately yearly MRI studies. The recurrent tumor showed progressive, interval growth, reaching a measurement of 3.2 × 3.6 cm in December 2005 (Figure 2), 9.2 × 3.4 cm in January 2008, and 11.0 × 8.9 cm in January 2012, at which time there was possible invasion into the right corpus cavernosum.

**Figure 3:**
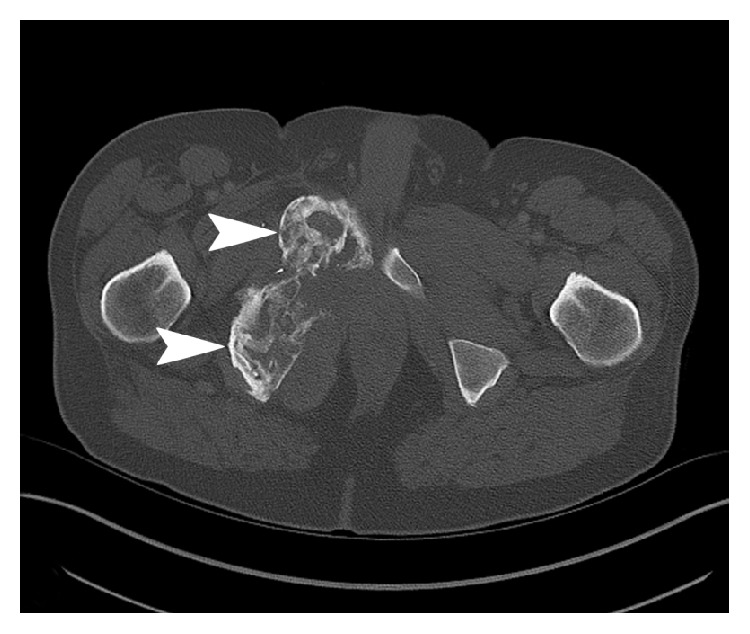
Axial CT of the pelvis (February 2015) showed extensive cortical irregularity and cystic spaces (arrows) of the right inferior pubic ramus (bone window algorithm).

**Figure 4:**
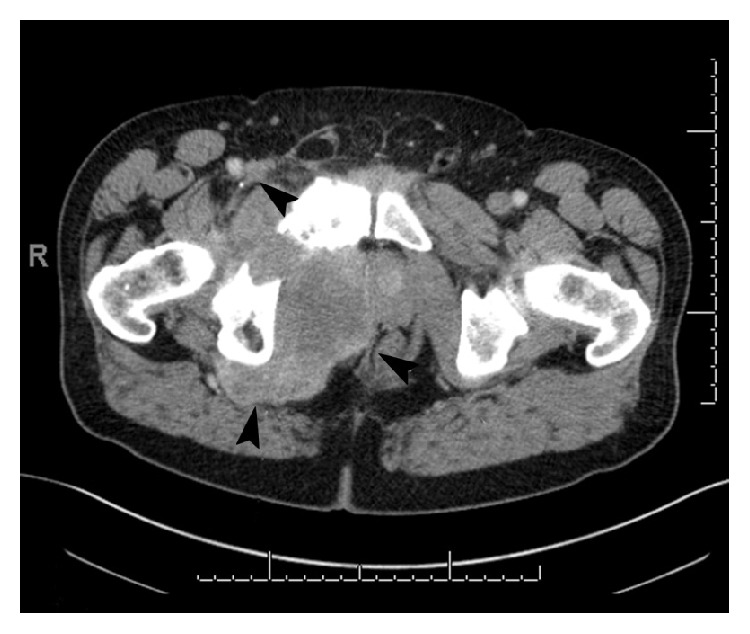
Axial CT of the pelvis (February 2015) showed severe interval enlargement of the soft tissue mass (arrows) surrounding the right inferior ramus (soft tissue algorithm).

The patient was started on denosumab (120 mg subcutaneous monthly) in July 2012. Imaging over the next 2 years showed stable disease, with possible slight decrease in size of the tumor. In November 2014, the patient developed osteonecrosis of the left mandible after tooth extraction, and denosumab was held for two doses. Denosumab was restarted in late December 2014 after presenting to clinic with severe right groin pain radiating inferiorly. Follow-up CT scan in February 2015 showed enlargement of the pelvic mass to 13.6 × 13.0 cm with extension into the ischioanal fossa and subsequent mass effect on the rectum, prostate, bladder base, and base of penis (Figures 3 and 4). The imaging findings were highly suggestive of sarcomatous transformation of the GCT. Multiple nodules were also now noted throughout the lungs. No additional potential primary tumor that could account for the lung nodules was identified on imaging, and metastases from a malignant GCT were suspected. Denosumab was discontinued.

**Figure 5:**
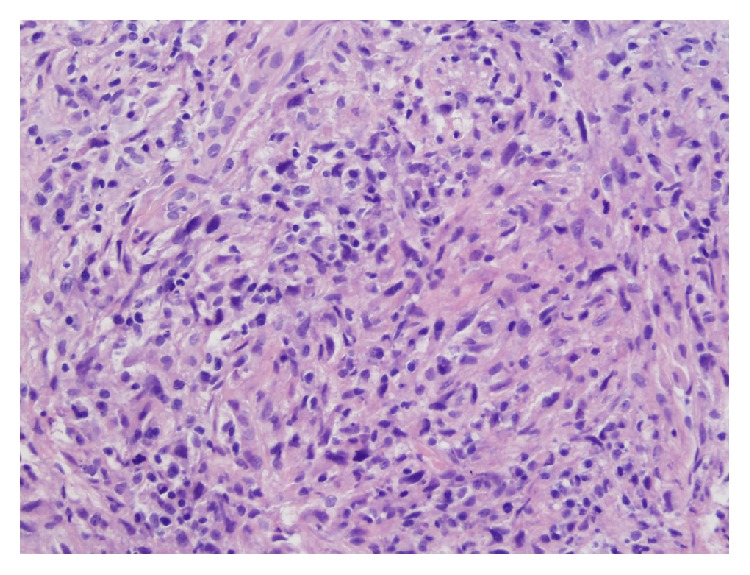
Biopsy of lung nodule revealed a sarcoma comprised of high grade spindle-shaped cells, consistent with metastatic sarcoma (hematoxylin and eosin stain, 20x).

**Figure 6:**
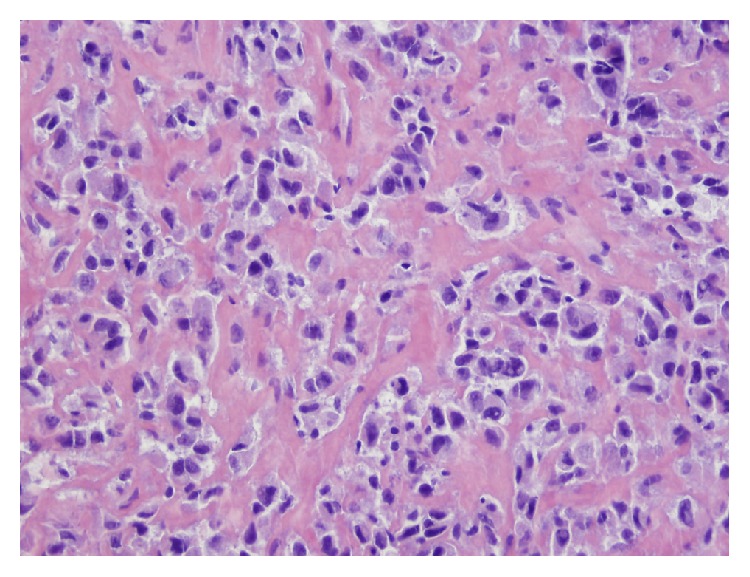
Biopsy of enlarging pelvic mass showed a sarcoma comprised of high grade round to epithelioid cells (hematoxylin and eosin stain, 40x).

CT guided biopsy of a right lower lobe nodule showed a high grade spindle cell sarcoma with nuclear atypia and low mitotic rate (up to 4 mitoses per high power field) (Figure 5). By immunohistochemistry, the tumor stained for smooth muscle actin (SMA) and p63, with focal S-100 protein staining. Subsequent CT guided biopsy of the pelvic mass revealed a high grade sarcoma with discohesive round to epithelioid cells that expressed SATB2 and weak SMA and lacked p63 (Figure 6). The morphology and IHC staining results were consistent with transformation to osteosarcoma.

Subsequent CT scan showed interval increase in size of the pelvic mass, an increase in the number and size of lung nodules, pulmonary lymphadenopathy, and a hepatic lesion suspicious of additional metastasis. The patient was started on doxorubicin and ifosfamide. CT scan showed slightly decreased primary tumor but an increased number of pulmonary nodules, two liver lesions, inguinal lymphadenopathy, and a probable 3.7 cm metastasis to the penis. He was then started on docetaxel and gemcitabine. He transferred care from our institution shortly thereafter.

### 2.2. Case  2

**Figure 7:**
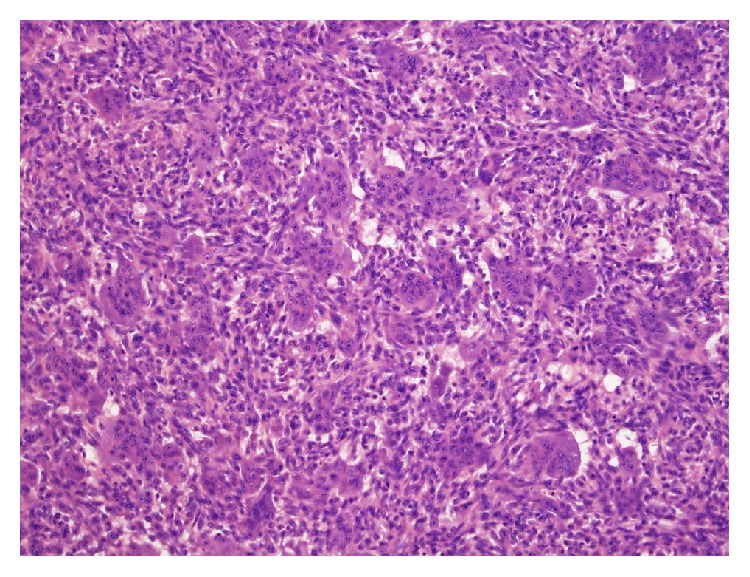
Histology of curettage specimen showed bland spindle cells and intermixed giant cells (hematoxylin and eosin stain, 20x).

A 49-year-old male presented in July 2007 with a six-month history of left knee pain and swelling. Radiographs from an outside institution showed a cystic lesion in the left femur condyle with sclerotic borders and expansion of surrounding bone. GCT was strongly suspected and the patient underwent curettage with PMMA packing in August 2007. Histologic evaluation confirmed giant cell tumor of bone (Figure 7).

**Figure 8:**
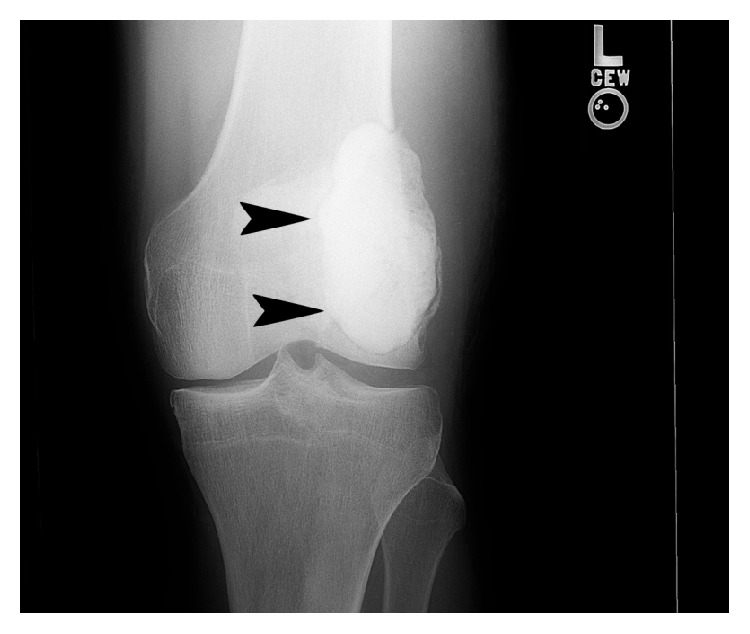
Frontal left knee radiograph (August 2007) demonstrates postsurgical curettage and packing with radiopaque polymethylmethacrylate (PMMA) of a well-defined, solitary, mixed lytic, and sclerotic lesion with a narrow zone of transition (arrows), located within the distal femoral metaphysis.

**Figure 9:**
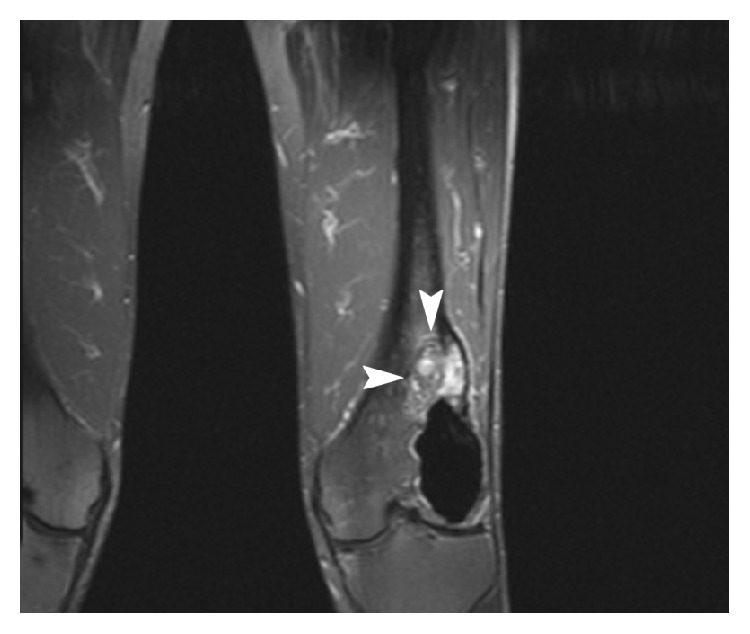
Coronal T1 postcontrast CT (November 2013) demonstrated a heterogeneously enhancing soft tissue mass (arrows) within the lateral aspect of the distal femoral condyle, proximal to the hypointense focus of PMMA.

The patient was followed postoperatively with radiographs of the knee. One month after surgery, a rim of lucency around the cement packing was noted (Figure 8). There was interval increase in the lucency until February 2009, when MRI showed an 8.15 × 3.9 × 1.8 cm area of marrow infiltration corresponding to the lucency, consistent with recurrence. There was minimal interval increase in size on MRI through November 2013, during which time the patient experienced some intermittent left knee pain, when an additional 2 × 1 cm mass was identified, along with mild periostitis along the lateral distal femur (Figure 9).

**Figure 10:**
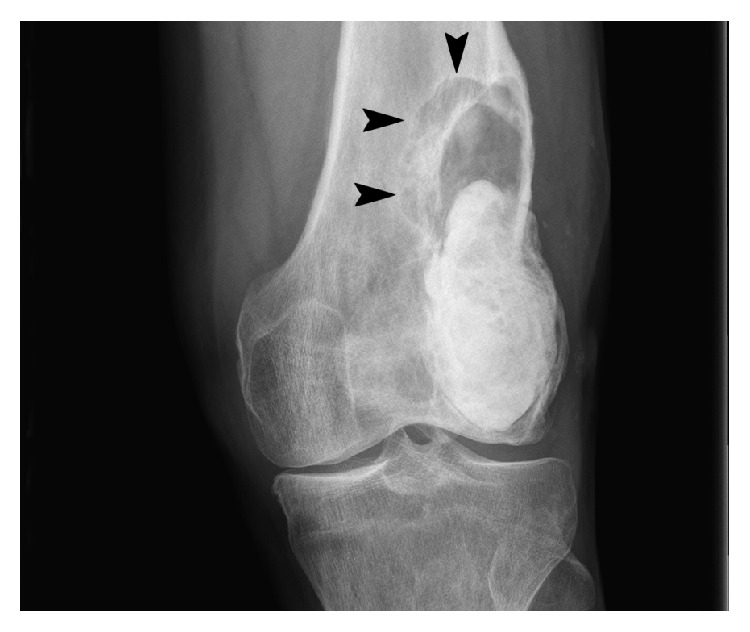
Frontal left knee radiograph (July 2014) demonstrated increased well-defined lytic focus (arrows) surrounding the radiopaque PMMA, suggestive of malignant transformation.

The patient was offered, and declined, both denosumab and surgery at that time. With continued knee pain and weakness, however, he opted for denosumab treatment in January 2014. He initially did well, denying pain except with running or other significant impact activities. But, by July 2014, he reported two months of significantly increasing knee pain and swelling. Radiographs showed continued increase in size of the lucency, measuring up to 5.0 cm, with periosteal reaction suggestive of impending pathologic fracture (Figure 10). Because of the intolerable pain, the patient opted for surgery. Due to the tumor's proximity to the bone cortex and articular surface, curettage was not possible and arthroplasty was scheduled. The patient underwent wide resection with endoprosthetic reconstruction in August 2014.

**Figure 11:**
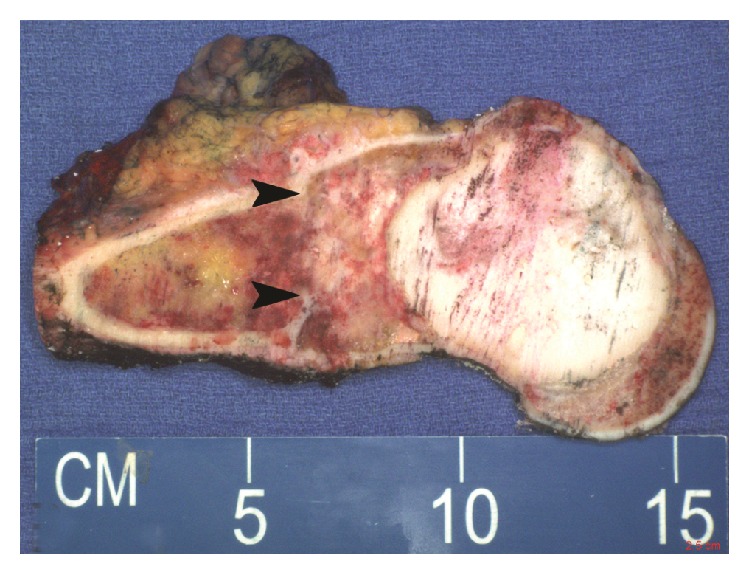
Distal femoral amputation demonstrated an ill-defined mass (arrows) proximal to the PMMA packing.

**Figure 12:**
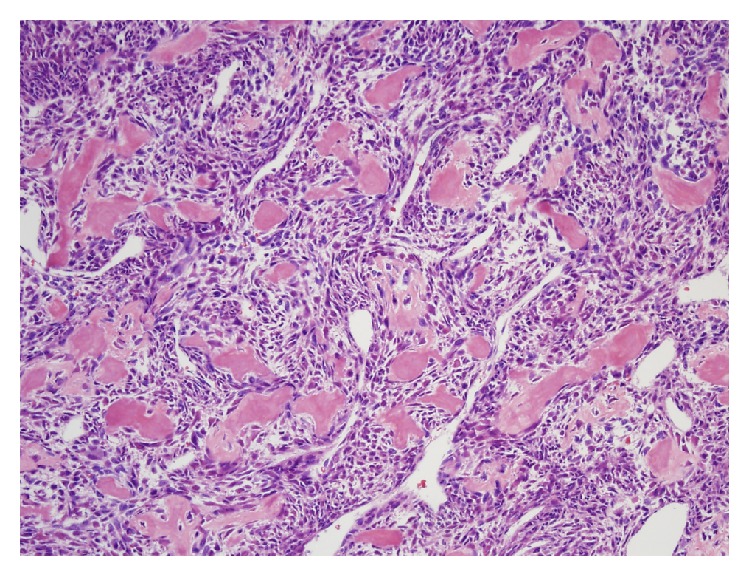
Histologic examination of area shown in Figure 11 showed atypical, hyperchromatic spindle cells and osteoid formation, consistent with osteosarcoma (hematoxylin and eosin stain, 20x).

The pathology specimen consisted of a 15 cm length of distal femur with minimal attached soft tissue and muscle and with an up to 4.0 cm ill-defined heterogeneous mass proximal to the PMMA (Figure 11). Histologic examination showed that the mass was comprised of pleomorphic osteoblastic cells in a fibrosarcomatous arrangement with bone, osteoid, and focal cartilage formation (Figure 12). Mitoses averaged 18 per high powered field. By immunohistochemistry, the tumor cells stained with SATB2, p53, and p63. Multiple soft tissue margins were positive. The findings were consistent with a high grade osteosarcoma arising from giant cell tumor. Cytogenetic analysis of tumor tissue revealed a complex karyotype that included loss of chromosome 17, a recurrent finding in osteosarcoma.

The patient was given four cycles of doxorubicin and cisplatin in September 2014 but developed skeletal metastases. He was then started on high dose methotrexate. He initially had stable disease but again progressed after several months, with development of pleural and additional skeletal metastases. He was then placed on gemcitabine and docetaxel but died soon after.

## 3. Discussion

Recently, the first case report of sarcoma arising in the setting of denosumab treatment for giant cell tumor of bone was published [14]. The patient was a 20-year-old female with a five-year history of GCT of the proximal tibia, initially treated with intralesional resection. The tumor recurred twice over the next 3.5 years and was treated both times with intralesional resection. After the second resection, she was treated with denosumab and presented one year later with a rapidly growing mass in the proximal tibia. Open biopsy revealed a high grade, mitotically active sarcoma with extensive necrosis. The patient subsequently underwent amputation.

Two Phase II trials have thus far evaluated the safety and efficacy of denosumab in the treatment of giant cell tumor of bone. In a study of 37 patients with recurrent or unresectable GCT who received 120 mg of denosumab monthly (and loading doses on days 8 and 15 of the first month), 30 of 35 patients with available follow-up data had tumor response, defined as elimination of at least 90% of giant cells on biopsy or no radiologic progression. Denosumab was well tolerated, with pain and nausea as the primary complaints. One patient developed high grade sarcoma arising in a GCT, found after an abnormal elevated human chorionic gonadotropin level and subsequent tumor resection. Another patient with recurrent GCT metastatic to lung developed malignant GCT eight months after discontinuing treatment [13].

A second Phase II trial utilizing the same doses and scheduling of denosumab as the first trial enrolled 282 patients with GCT into three cohorts: those with surgically unsalvageable disease (*n* = 170); those with salvageable disease with planned surgery (*n* = 101); and a third cohort (*n* = 11) that included patients from the original Phase II study of 37 patients. Ninety-six percent of patients in the surgically unsalvageable disease cohort showed no progression. Only 26 patients in the planned surgery cohort ultimately required surgery, 16 of these with a less morbid surgery than originally planned. Again, denosumab was well tolerated, with pain, nausea, and fatigue as the primary complaints. Hypophosphatemia, hypocalcemia, infection, and osteonecrosis of the jaw were noted in a handful of patients. Two cases of sarcoma arising in GCT were recorded, though it is unclear in which cohort(s) these patients were. One case was suspected to have been present but unrecognized prior to treatment. The other case arose during the study. The investigators did not believe in a connection between denosumab and sarcomatous transformation of GCT. Interestingly, a case of thyroid carcinoma with high grade sarcoma was also reported, arising in an area of previous radiation [12]. At our institution, only six patients with giant cell tumor of bone have been treated with denosumab. Of these, four received more than six months of therapy. Two of these patients with a longer duration of denosumab therapy are the two patients presented in this paper. One of the remaining two patients, a 30-year-old woman, has recently developed a 1 cm soft tissue mass near the site of her original tumor following three recurrences in the radius over 60 months and following 36 months of denosumab therapy. That mass has not yet been biopsied.

The potential relationship between, and mechanism of, sarcomatous transformations of GCT during denosumab therapy is unclear due at least in part to the limited published data on this population. In vivo and in vitro studies of the effect of denosumab on GCT at the cellular level show loss of giant cells, usually a reduction in the neoplastic stromal cells with reduced RANKL expression and proliferation, and reactive and woven bone and/or osteoid formation [13, 15, 16]. Proliferation of spindle cells with reactive bone and osteoid formation has also been reported [17, 18].

Patients taking denosumab for GCT likely represent a subset that are at higher baseline risk for sarcomatous transformation, often having a long-standing history of disease with multiple recurrences and treatments. More extensive studies of the side effects of denosumab in patients with osteoporosis [19] and cancer [20–22] have not noted any increase in the risk of sarcoma, though denosumab dosing is lower in the former group and long-term follow-up is not consistent in the latter group. At our institution, 14 patients with giant cell tumor of bone have had recurrences (from a total of 40 patients with GCT with 4 patients lost to follow-up <6 months after treatment); of these, two patients have had malignant transformation: the two patients reported herein who also represent half of the patients treated with denosumab for at least six months (average follow-up: 83 months, range of 6–154). Additional controlled studies and long-term follow-up are needed before more definitive conclusions can be drawn regarding denosumab treatment and sarcomatous transformation of giant cell tumor of bone.
